# Inducible Sterilization of Zebrafish by Disruption of Primordial Germ Cell Migration

**DOI:** 10.1371/journal.pone.0068455

**Published:** 2013-06-27

**Authors:** Ten-Tsao Wong, Paul Collodi

**Affiliations:** Department of Animal Sciences, Purdue University, West Lafayette, Indiana, United States of America; National University of Singapore, Singapore

## Abstract

During zebrafish development, a gradient of stromal-derived factor 1a (Sdf1a) provides the directional cue that guides the migration of the primordial germ cells (PGCs) to the gonadal tissue. Here we describe a method to produce large numbers of infertile fish by inducing ubiquitous expression of Sdf1a in zebrafish embryos resulting in disruption of the normal PGC migration pattern. A transgenic line of zebrafish, *Tg(hsp70:sdf1a-nanos3, EGFP)*, was generated that expresses Sdf1a under the control of the heat-shock protein 70 (*hsp70*) promoter and *nanos3* 3?UTR. To better visualize the PGCs, the *Tg(hsp70:sdf1a-nanos3, EGFP)* fish were crossed with another transgenic line, *Tg(kop:DsRed-nanos3)*, that expresses DsRed driven by the PGC-specific *kop* promoter. Heat treatment of the transgenic embryos caused an induction of Sdf1a expression throughout the embryo resulting in the disruption of their normal migration. Optimal embryo survival and disruption of PGC migration was achieved when transgenic embryos at the 4- to 8-cell stage were incubated at 34.5°C for 18 hours. Under these conditions, disruption of PGC migration was observed in 100% of the embryos. Sixty-four adult fish were developed from three separate batches of heat-treated embryos and all were found to be infertile males. When each male was paired with a wild-type female, only unfertilized eggs were produced and histological examination revealed that each of the adult male fish possessed severely under-developed gonads that lacked gametes. The results demonstrate that inducible Sdf1a expression is an efficient and reliable strategy to produce infertile fish. This approach makes it convenient to generate large numbers of infertile adult fish while also providing the capability to maintain a fertile brood stock.

## Introduction

Efficient aquaculture production is essential to meet the growing demand for aquatic food species. From 2002 to 2010 annual aquaculture production increased from 36.8 to 60 million tons with a value of $119 billion [1]. As our dependence continues to shift away from wild populations towards artificially propagated aquatic species, continual optimization of aquaculture methods in an environmentally sustainable fashion will be necessary to maximize food production. Also, reliable strategies must be developed for the genetic containment of farmed fish to minimize environmental risk due to accidental release of the cultured species.

The most effective bio-containment strategy for large-scale commercial aquaculture operations is the use of infertile farmed fish. Since infertile fish that escape aquaculture containment will not be able to reproduce with each other or with wild fish, their spread and genetic mixing with local fish populations are prevented. Sterilization also increases the fish growth rate by enhancing the conversion of food energy to muscle growth instead of gonad and germ cell development resulting in more efficient aquaculture production [2]. In this paper we describe an efficient and reliable strategy to generate large populations of infertile fish by disrupting the normal migration of primordial germ cells (PGCs) to the developing gonad in the fish embryo.

PGCs are a population of cells in the embryo that give rise to the eggs and sperm of the adult. In fish, PGCs are specified during early development by the incorporation of maternally-derived germ plasm [3], [4]. The PGCs then migrate to the developing gonad by following a gradient of the chemokine, stromal-derived growth factor (Sdf1a) [5], [6]. Disruption of the Sdf1a signaling pathway prevents normal PGC migration in the fish embryo [5], [7]. In this study, we demonstrate that induced expression of Sdf1a in the zebrafish embryo prevents the PGCs from responding to the endogenous Sdf1a gradient resulting in mis-migration of the PGCs and the development of a sterile fish. Inducible Sdf1a expression provides a convenient and economical strategy to generate large numbers of sterile adult fish while also maintaining a fertile (non-induced) brood stock population.

## Materials and Methods

### Animals and Ethics

Zebrafish were maintained and staged as previously described [8]. All of the experimental procedures and protocols described in this study were approved by the Purdue University Animal Care and Use Committee and adhered to the National Research Council’s Guide for Care and Use of Laboratory Animals.

### Plasmid Construction

To clone the cDNAs that encode zebrafish Sdf1a, the primers Fwd1∶5?-CTCTTCTTCACGGTACCAACATGGATCTCA-3? and Rev1∶5?-TTCCTTGTCATGCGGCCGCCATCTTAGA-3? were designed to amplify cDNA from zebrafish embryonic cDNA using Advantage® 2 PCR Kit (Clontech). The PCR program was 95°C (1 min), 35 cycles of 94°C (10 sec)/60°C (10 sec)/68°C (1 min) and 68°C (6 min). To clone the *kop* promoter fragment, the forward primer Fwd2∶5?-TCTGTCTTCTGGACACAATGCCTCTG -3? and reverse primer Rev2∶5?-TGAATGGATGTATCTGTGAATGACATTTTTG-3? were used with zebrafish testicular genomic DNA template and the PCR program was: 95°C (1 min), 30 cycles of 94°C (10 sec)/68°C (6 min) and 68°C (10 min) using Advantage® Genomic LA Polymerase Mix (Clontech). The PCR products were first cloned into pGEM-T-easy vector (Promega) and the sequence of each clone was verified. The zebrafish heat-shock protein70 (*hsp70*) promoter fragment (kind gift from Dr. Warren James, Penn State Erie [9]), and the *nanos3* 3?UTR [10] were incorporated 5? and 3? respectively flanking either *sdf1a or EGFP* to generate *hsp70-sdf1a-nanos3 3?UTR* and *hsp70-EGFP-nanos3 3?UTR* expression constructs. These two constructs were ligated together and sub-cloned into a modified Tol2pA [11] vector resulting in the expression cassette being flanked with Tol2 transposon sites to enhance the efficiency of genomic integration. The resulting plasmids were designated pHsp-SE. The verified *kop* promoter fragment was digested by XhoI and EcoRI to generate a 3.8 kb fragment (64926 to 61158 in NW_003335885) that was assembled 5? of *DsRed* with *nanos3* 3?UTR to generate the *kop-DsRed-nanos3 3?UTR* expression construct flanked with Tol2 transposon sites as described above to generate pKop-DsRed.

### Production of Transgenic Fish

To produce transgenic fish, 1 to 2 nl of a solution containing 7.5 ng/µl Tol2 RNA and 25 ng/µl of either pHsp-SE or pKop-DsRed was injected into 1- to 2-cell stage embryos. The embryos were raised to adults and the male founders were identified by screening sperm samples for germline transmission of the construct. Sperm was collected from each male and analyzed by PCR using *hsp70* forward primer Fwd3∶5?-ACATGTGGACTGCCTATGTTCATC-3? and *sdf1a* reverse primer Rev1 or EGFP reverse primer Rev3∶5?-GTGCTCAGGTAGTGGTTGTC3-3? for pHsp-SE injected fish, and *kop* forward primer Fwd4∶5?-ATCTGCTCCGTAAAAATGTGCT-3? and DsRed reverse primer Rev4∶5?-AGCCCATGGTCTTCTTCTGCATCA-3? for pKop-DsRed injected fish. In our initial experiments, we identified 12 *Tg(hsp70:sdf1a-nanos3, EGFP)* and 9 *Tg(kop:DsRed-nanos3)* founder fish. Three founders of each line were chosen for further analysis and out-crossing to wild-type females to produce the stable lines. Genomic PCR screening was performed to identify F1 and F2 offspring that carried *hsp70-sdf1a* and *hsp70-EGFP* or *kop-DsRed* using the primers listed above. Tissue obtained by fin-clipping or from 5 dpf individual embryos was used for genomic DNA extraction [12].

### Heat Induction of *sdfl1a* Expression

To induce *sdf1a* expression, *Tg(hsp70:sdf1a-nanos3, EGFP)* embryos at the 4–8 cell stage (1 hpf) were incubated at different temperatures ranging from 28.5 to 36.5°C for 18 hours. To visualize the effect of heat-induced Sdf1a expression on PGC migration, female *Tg(kop:DsRed-nanos3)* that carried maternal DsRed labeled PGCs were crossed with *Tg(hsp70:sdf1a-nanos3, EGFP)* males by in?vitro fertilization [8]. The number and location of DsRed-positive PGCs present in each embryo was examined and counted at 2 dpf using a Nikon Eclipse TE200 fluorescence microscope (Nikon, Japan) equipped with a RT Slider digital camera (Spot Imaging Solution, Sterling Heights, MI, USA).

### Whole-mount in situ Hybridization

Zebrafish *sdf1a* cDNA-containing plasmid was linearized and used as templates for anti-sense and sense digoxigenin (DIG) labeled riboprobe synthesis (Roche, Indianapolis, IN) according to the manufacturer’s instructions. Whole-mount in situ hybridization was used to detect *sdf1a* expression according to a published protocol [13]. Fixed and methanol-preserved embryos were rehydrated and treated with proteinase K (10 µg/ml) for 10 min. Samples were post-fixed for 30 min in 4% paraformaldehyde, treated with cold acetone for 8 min, and prehybridized for 3 hours at 65°C in hybridization buffer (50% formamide, 5× SSC, 0.1% CHAPS, 5 mM EDTA, 0.1% Tween 20, 1 mg/ml yeast RNA, and 50 mg/ml heparin). DIG-labeled anti-sense or sense *sdf1a* riboprobes were added into hybridization buffer at a final concentration of 1 µg/ml. After incubation at 65°C for overnight, samples were washed with 50% formamide and 2× SSC at 60°C, 1× SSC at 60°C, and 0.2× SSC at 65°C. Alkaline phosphatase (AP) coupled anti-DIG antibody (Roche) at 150 mU/ml was used to detect DIG in the samples. After color development using Purple AP substrate (Roche), samples were examined under a light microscope (Nikon Eclipse TE200) equipped with a RT Slider digital camera (Spot Imaging Solution).

### Histology

Zebrafish were euthanized in 0.016% tricaine (ethyl-3-aminobenzoate methanesulfonic acid) (Sigma-Aldrich) solution in water, and gonads were removed and fixed with Bouin fixative at 4°C overnight. After two rinses in phosphate buffered saline (PBS), the fixed gonads were processed through successive ethanol treatments (50%, 70%, 95%, and 100%) followed by two xylene treatments and embedded in paraffin. The serial 5 µm paraffin sections were prepared, using an American Optical model 820 microtome (American Optical Corporation). For histology, the sections were stained with hematoxylin-eosin and examined by light microscopy.

### Statistical Analysis

Data were presented as the mean and standard deviation. For statistical analysis one-way ANOVA was applied followed by a Bonferroni–Dunn test using the SAS program. The significance was accepted at *p<*0.05.

## Results

### Production of *Tg(hsp70:sdf1a-nanos3, EGFP)* zebrafish

**Figure 1:**
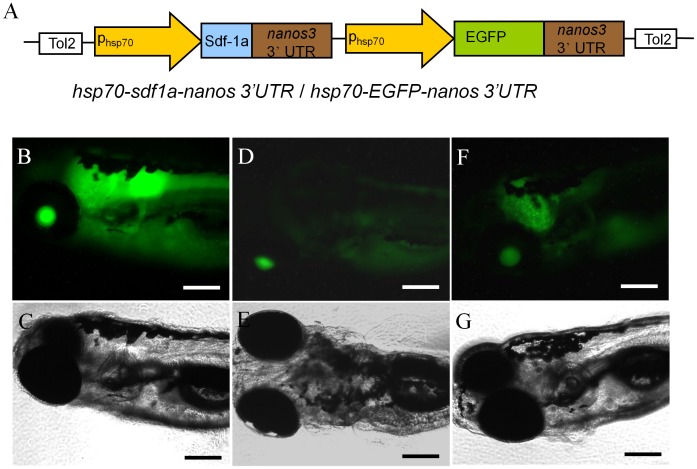
Generation of *Tg(hsp70:sdf1a-nanos3, EGFP)* zebrafish. (A) Diagram of the plasmid construct used to generate the transgenic line. A 1.5-kb fragment of the *hsp70* promoter controls expression of zebrafish *sdf1a* and *EGFP*. Three different EGFP expression patterns were observed in F1 offspring obtained from different founder fish. (B,C) strong ubiquitous expression, (D,E) weak expression throughout the body, (F,G) strong expression in the brain and weak in the body. The strongly expressing larvae were raised to sexual maturity and used to establish the homozygous line. Scale bar = 200 µm.

**Figure 2:**
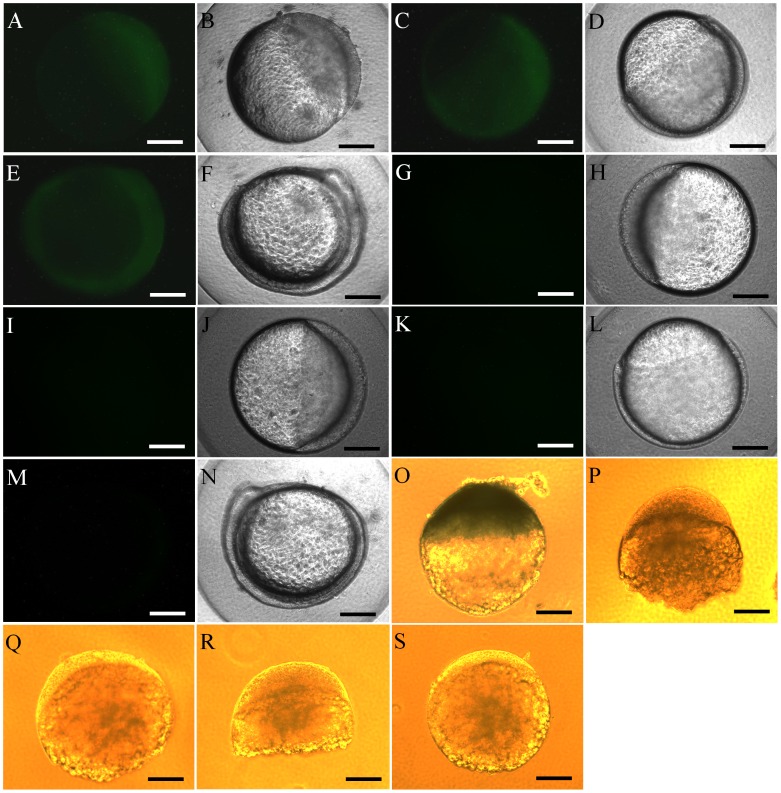
Heat induced expression of EGFP and *sdf1a* in *Tg(hsp70:sdf1a-nanos3, EGFP)* embryos. (A,B) Expression of EGFP was first observed at 30% epiboly in the embryos treated at 35.5°C, (C, D) 50% epiboly in the embryos treated at 34.5°C and (E, F) 2–5 somite in the embryos treated at 32.5°C. (G–N) EGFP expression was not detected by the 2 to 5 somite-stage in the embryos treated at 30.5 or 28.5°C. (O) The ubiquitous expression of *sdf1a* (dark blue) was quickly (less than 2 hours of staining) detected in heat-treated transgenic embryos started from late blastula stage. No obvious signal was seen in (P) non-heat-treated transgenic embryos, (Q) heat-treated wild-type embryos or (R) non-heat-treated wild-type embryos using the same staining condition. (S) No signal was detected when *sdf1a* sense riboprobe was used in heat-treated transgenic embryos as controls. Scale bar = 200 µm.

A transgenic line of zebrafish, *Tg(hsp70:sdf1a-nanos3, EGFP)*, was produced in which the embryos express Sdf1a under the control of the inducible *hsp70* promoter. A 1.5 kb fragment of the zebrafish *hsp70* promoter [17] and the zebrafish *nanos3* 3?UTR [10] were used to control inducible Sdf1a expression in the embryos. To monitor the level of inducible expression, the transgenic fish carried EGFP also under the control of the *hsp70* promoter and *nanos3* 3?UTR. A diagram of the construct used to generate the transgenic line is shown in Fig. 1A. In the absence of heat treatment, EGFP expression was not observed in the F1 embryos through 2 days post fertilization (dpf). Fluorescence was first detected at 3 dpf, and by 6 dpf different EGFP expression patterns were observed in F1 offspring produced from different founders. Some embryos exhibited strong EGFP expression throughout the body (Fig. 1B,C) while the remaining embryos expressed weaker and non-uniform expression (Fig. 1D–G). The strongly expressing larvae were raised to sexual maturity and used to establish the homozygous *Tg(hsp70:sdf1a-nanos3, EGFP)* line. Heat-induction of the *hsp70* promoter was examined by exposing the transgenic embryos at the 4- to 8-cell-stage (1 hpf) to temperatures ranging from 28.5 to 36.5°C for 18 hours and monitoring the expression of EGFP. Treatment of embryos to temperatures up to 34.5°C did not significantly affect survival while temperatures of 35.5°C and 36.5°C resulted in approximately 75 and 100% mortality, respectively. Initial induction of EGFP expression was temperature dependent, first being detected at 30% epiboly in embryos treated at 35.5°C and at 50% epiboly in embryos treated at 34.5°C compared to the 2–5 somite stage in the 32.5°C treated group (Fig. 2A–F). Treatment temperatures of 28.5 and 30.5°C did not induce EGFP expression up through the 2–5 somite stage (Fig. 2G–N). Results from whole-mount in situ hybridization demonstrated that heat-treatment of transgenic embryos induced strong ubiquitous expression of *sdf1a* that was quickly (less than 2 hours of staining) detected in late blastula stage (Fig. 2O). No obvious signal was seen in non-heat-treated transgenic embryos, heat-treated wild-type embryos or non-heat-treated wild-type embryos using the same staining condition (Fig. 2P–R).

### Heat-induced Sdf1a Expression Results in Improper PGC Migration

**Figure 3:**
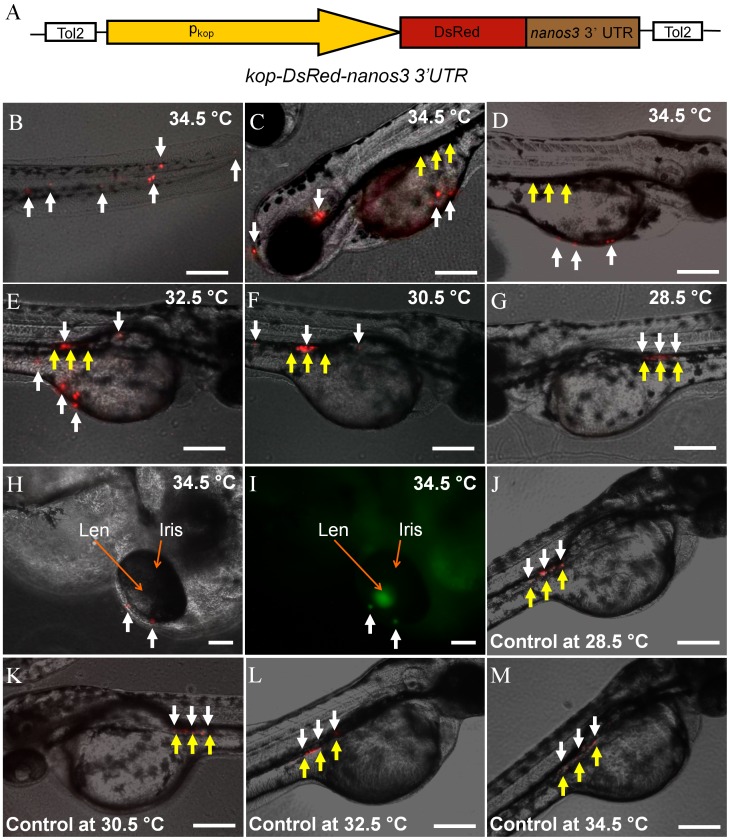
Disruption of PGC migration in heat-treated *Tg(hsp70:sdf1a-nanos3, EGFP)/Tg(kop:DsRed-nanos3)* embryos. (A) Diagram of the plasmid construct used to generate the *Tg(kop:DsRed-nanos3)* zebrafish. A 3.8 kb fragment of the zebrafish *kop* promoter and *nanos3* 3?UTR control PGC-specific DsRed expression. (B–E) Photomicrographs showing PGC migration in heat-treated *Tg(hsp70:sdf1a-nanos3, EGFP)/Tg(kop:DsRed-nanos3)* embryos. In embryos treated at 34.5°C at 2dpf the majority of ectopically located PGCs were observed in (B) the tail, (C) the head and (D) along the outer surface of the yolk sac. (E) Fewer ectopic PGCs were observed in the embryos treated at 32.5°C and (F, G) at 30.5 and 28.5°C the majority of PGCs migrated to the gonadal region. (H, I) Mis-migrated PGCs (red) also express EGFP. (J–M) No significant disruption of PGC migration was found when embryos of *Tg(kop:DsRed-nanos3)* were heat-treated at temperatures ranging from 28.5 to 34.5°C for 18 hours. White arrows: DsRed-expressing PGCs; yellow arrows: gonadal region. Scale bar = 200 µm.

Zebrafish PGCs begin their migration to the developing gonad at approximately 5 to 6 hpf [14]. To determine if induced Sdf1a expression disrupts PGC migration, the transgenic embryos were heat-treated at 1 hpf as described above and PGC movement was monitored. The PGC population was visualized by crossing *Tg(hsp70:sdf1a-nanos3, EGFP)* male fish with *Tg(kop:DsRed-nanos3)* females. The *Tg(kop:DsRed-nanos3)* embryos express DsRed specifically in the PGCs under the control of the *kop* promoter [15] and zebrafish *nanos3* 3?UTR [10]. A diagram of the construct used to generate the *Tg(kop:DsRed-nanos3)* fish is shown in Fig. 3A. The *Tg(hsp70:sdf1a-nanos3, EGFP)/Tg(kop:DsRed-nanos3)* embryos were incubated at temperatures ranging from 28.5 to 34.5°C for 18 hours beginning at 1 hpf and the location of the DsRed-positive PGCs was examined at 2 dpf. The results revealed that heat treatment at temperatures of 32.5°C or 34.5°C resulted in mis-migration of the PGCs to ectopic locations including the tail, head and yolk sac (Fig. 3B–E). Disruption of PGC migration correlated with the temperature of heat treatment since approximately 98% of the PGCs were identified at ectopic locations at 34.5°C compared to 70% at 32.5°C. PGC migration was only slightly affected when the embryos were incubated at 30.5°C and completely normal at 28.5°C (Fig. 3F, G). The mis-migrated PGCs also expressed EGFP that, however, was not able to be clearly identified due to the strong ubiquitous expression of hsp70-EGFP in most parts of body. Their EGFP expression could be seen only when they mis-migrated to the area, such as eye iris (Fig. 3H, I), where has no hsp70-EGFG expression. When embryos of *Tg(kop:DsRed-nanos3)* were heat-treated at temperatures ranging from 28.5 to 34.5°C for 18 hours, no significant disruption of PGC migration was found (Fig. 3J–M).

### Heat-treatment of *Tg(hsp70:sdf1a-nanos3, EGFP)* Embryos Results in the Development of Sterile Adult Fish

**Figure 4:**
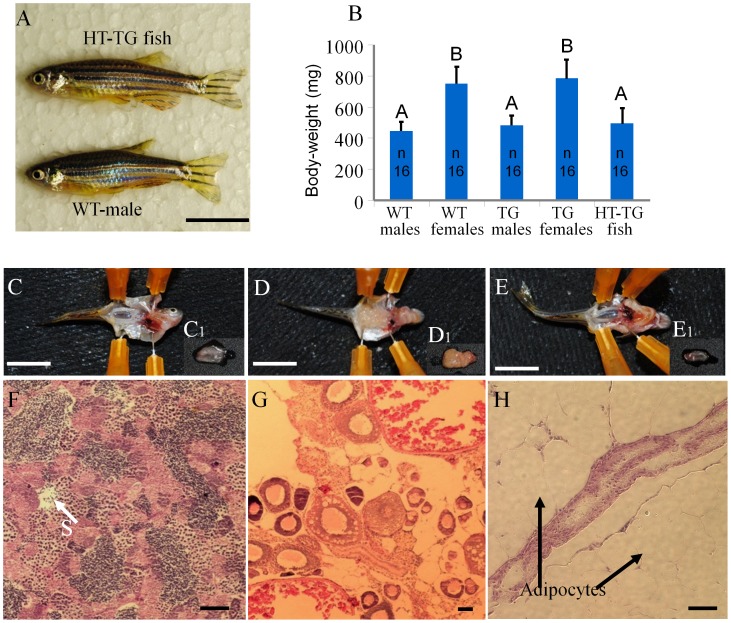
Heat-treated transgenic embryos developed into infertile male adults. (A) No difference in appearance or overall size was observed between adult transgenic fish that developed from heat treated embryos and wild-type male. (B) No significant difference in body-weight of 3.5-month-old fish (n = 16 by random sampling) among heat-treat transgenic males, untreated transgenic males and wild-type males, and between the transgenic females and wild-type females. Data shared the same letter (A or B) are not significantly different from each other. Examination of gonadal tissue revealed that (C) A well-developed testis, C1, of untreated male transgenic fish. (D) A well-developed ovary, D1, of untreated female transgenic fish. (E) The gonads of heat-treated transgenic fish developed into a thin filament-like tissue, E1, surrounded by adipocytes. Photomicrograph showing (F) active spermatogenesis of the testis of untreated male transgenic fish, (G) a well-developed ovary with oocytes at different developmental stages of untreated female transgenic fish. (H) The gonad of heat-treated transgenic fish appears to be under-developed and surrounded with large amount of adipocytes without advanced gonadal structure or germ cells. WT: wild type; TG: transgenic; HT: heat treated. Scale bar: 1 cm for A, C–E and 50 µm for F–H. S: spermatozoa.

Homozygous *Tg(hsp70:sdf1a-nanos3, EGFP)* embryos that were heat induced at 34.5°C for 18 hours beginning at the 4- to 8-cell stage were allowed to develop to adults and the fertility of the fish was examined. A total of 64 fish (Table 1), obtained from 3 separate batches of treated embryos, were examined. All of the fish were found to be males that exhibited no observable difference in appearance from wild type males (Fig. 4A, B). The transgenic male fish that were heat-treated as embryos were paired with wild-type females and at least 3 batches of eggs were collected from each pair. Although the males were able to induce wild-type female fish to release eggs in the spawning chamber, none of the eggs were successfully fertilized. Also no sperm could be expressed from the males by gently pressing on their abdomen. Dissections were performed to examine gonad development in the heat-treated transgenic fish and non-heat-treated transgenic control fish. The results revealed that testes (Fig. 4C,C1) and ovary (Fig. 4D,D1) development was normal in the non-heat-treated transgenic fish while gonad development was absent in the heat-treated individuals except for a thin filament of connective tissue in the gonadal region (Fig. 4E, E1). Histological examination of the partially formed gonad revealed the absence of gametogenesis. In contrast, the non-heat-treated transgenic male and female fish possessed fully formed gonads and active gametogenesis (Fig. 4F–H). The non-heat-treated fish were successfully bred to propagate the *Tg(hsp70:sdf1a-nanos3, EGFP-nanos3)* line. When wild-type embryos were treated at 34.5 or 35.5°C for 18 hours, they developed into normal males and females despite the low survival rate in the 35.5°C treated group (Table 1).

**Table 1:** Results from heat-treatment experiments on 1 hpf embryos of *Tg(hsp70:sdf1a-nanos3, EGFP)* at 34.5°C (H1-3), wild-type fish at 34.5°C (C1) or wild-type fish at 35.5°C (C2) for 18 hours.

Exp. groups	Number ofembryos treated	Number and (%) ofembryos survivedafter treatment	Number and (%) of fish surviving to adulthood	Number ofinfertile fishobtained	Number of fertile fish obtained
H1	29	27 (93.1%)	23 (79.3%)	23	0
H2	27	26 (96.3%)	20 (74.1%)	20	0
H3	26	23 (88.5%)	21 (80.8%)	21	0
Subtotal	82	76 (92.6%)	64 (78%)	64	0
C1*	50	47 (94%)	37 (74%)	0	24M**/13F
C2*	50	12 (24%)	8 (16%)	0	5M/3F

M: male. F: female.

* Anatomical examination of gonad development.

** 2 males were found to have developed only one side of testis.

## Discussion

As the U.S. Food and Drug Administration moves closer to approving the first genetically modified fish for aquaculture production [16], it is imperative that efficient and cost-effective methods of genetic containment are developed. This study demonstrates that forced expression of Sdf1a in the fish embryo during early development is an effective strategy to disrupt PGC migration and produce large populations of infertile fish. Our technology includes a heat-shock inducible promoter and a targeting strategy that made use of the 3?UTR of *nanos3* gene to specifically prolong Sdf1a expression in PGCs. *nanos3* is a germ cell specific gene whose 3?UTR carries a specific signal to direct [17], [18], [19] and prolong [10] the expression of Nanos3 in PGCs. The use of *nanos3* 3?UTR was designed to generate an autocrine action that directs the PGCs to produce and release their own Sdf1a that binds to their cell surface Sdf1a receptors, thereby preventing the PGCs from recognizing the endogenous Sdf1a gradient and migrating to the developing gonad. However, expression of Sdf1a was not specifically restricted in the PGCs because of the use of *hsp70* promoter that generated strong ubiquitous expression of Sdf1a after induction. Besides the autocrine action of Sdf1a controlled by *nano3* 3?UTR, the ubiquitous expression of Sdf1a driven by *hsp70* promoter also disrupted the endogenous Sdf1a gradient. As a result, we were not able to evaluate the specific effect of the use of *nanos3* 3?UTR in our method despite the technology is able to produce infertile fish. Our approach has the advantage of efficiently inducing infertility without affecting the survival or other physiological characteristics of the fish. Additionally, the use of an inducible promoter such as *hsp70* to control Sdf1a expression makes it convenient to produce a fertile brood stock population by simply omitting the induction treatment of the embryos. In our study, non-heat-treated embryos developed to produce fertile adults that could be bred multiple times to maintain the transgenic line.

The use of heat induction to produce infertile fish eliminates the need for chemical or hormonal treatment to induce sterility in the fish or to rescue fertility for brood stock production. Other methods to generate infertile fish have been developed including the manipulation of chromosome set by triploidization or interspecies hybridization [20], [21], [22], disruption of gonadotropin releasing hormone (Gnrh) signaling [23], [24], [25] and genetic ablation of the germ cells [26], [27]. Although these methods are effective in producing infertile fish, each has a disadvantage of either being not 100% reliable, impractical for large-scale aquaculture operations or not conducive to the generation of a fertile brood stock population [22], [23], [24], [26], [27], [28].

The mechanism of PGC migration is highly conserved across species. The involvement of Sdf1 as a directional cue for PGC migration has been demonstrated in multiple fish species as well as *Xenopus*, birds and mice [5], [6], [29], [30], [31], [32]. Therefore, it is reasonable to expect that the strategy to block PGC migration by interfering with Sdf signaling will be effective in all species of farmed fish. The Sdf1a amino acid sequence is highly conserved across fish species with 75% to 95% homology between zebrafish, medaka, carp, catfish, tilapia and salmon based on the comparison of published sequence data. It is likely that recombinant Sdf1a from one species will be active in disrupting PGC migration in multiple related species of farmed fish which would eliminate the need to clone *sdf* cDNAs from each farmed species.
